# The Mitogen-Activated Protein Kinase (MAPK) Pathway: Role in Immune Evasion by Trypanosomatids

**DOI:** 10.3389/fmicb.2016.00183

**Published:** 2016-02-24

**Authors:** Mercedes Soares-Silva, Flavia F. Diniz, Gabriela N. Gomes, Diana Bahia

**Affiliations:** ^1^Departamento de Biologia Geral, Instituto de Ciências Biológicas, Universidade Federal de Minas GeraisMinas Gerais, Brazil; ^2^Departamento de Microbiologia, Imunologia e Parasitologia, Escola Paulista de Medicina, Universidade Federal de São PauloSão Paulo, Brazil

**Keywords:** MAP kinase, *Trypanosoma cruzi*, *Leishmania*, immune evasion of parasites, cellular signaling

## Abstract

*Leishmania* spp. and *Trypanosoma cruzi* are the causative agents of leishmaniasis and Chagas disease, respectively, two neglected tropical diseases that affect about 25 million people worldwide. These parasites belong to the family Trypanosomatidae, and are both obligate intracellular parasites that manipulate host signaling pathways and the innate immune system to establish infection. Mitogen-activated protein kinases (MAPKs) are serine and threonine protein kinases that are highly conserved in eukaryotes, and are involved in signal transduction pathways that modulate physiological and pathophysiological cell responses. This mini-review highlights existing knowledge concerning the mechanisms that *Leishmania* spp. and *T*. *cruzi* have evolved to target the host’s MAPK signaling pathways and highjack the immune response, and, in this manner, promote parasite maintenance in the host.

## Introduction

*Leishmania* spp. and *Trypanosoma* *cruzi* are protozoan parasites of the Trypanosomatida order (Kent, 1980) and Trypanosomatidae family (Doflein, 1901). They are the etiological agents of leishmaniasis and Chagas disease, respectively, and are transmitted by the bite of infected sandflies (leishmaniasis) or through triatomine bug feces (Chagas disease). Both *Leishmania* spp. and *T. cruzi* have complex life cycles comprising diverse developing forms that alternate between the insect vector and the vertebrate host. *Leishmania* spp. promastigotes and amastigotes preferentially infect phagocytic cells of vertebrates, while *T. cruzi* metacyclic trypomastigotes, blood trypomastigotes and amastigotes are able to infect both phagocytic and non-phagocytic cells (Tanowitz et al., 1992; Alexander et al., 1999; Ferreira et al., 2012).

Although the persistence of *Leishmania* spp. and *T. cruzi* within a host depends on several factors, the manipulation of host signal transduction pathways involved in the modulation of the immune response is probably one of the most commonly used mechanisms by parasites. In this mini-review, we will focus on the mechanisms that *Leishmania* spp. and *T. cruzi* use to subvert mitogen-activated protein kinase (MAPK) signaling pathways—more specifically, extracellular-signal-regulated kinase (ERK), and p38 MAPK—that are highly relevant in the context of the regulation of the immune response against intracellular parasites.

## MAPK Pathways

Mitogen-activated protein kinases are protein kinases that phosphorylate their own dual serine and threonine residues (autophosphorylation), or those found on their substrates, to activate or de-activate their target (Johnson and Lapadat, 2002; Peti and Page, 2013). Accordingly, MAPKs regulate important cellular processes such as proliferation, stress responses, apoptosis and immune defense (Dong et al., 2002; Liu et al., 2007; Arthur and Ley, 2013). MAPKs are ubiquitously expressed and evolutionarily conserved in eukaryotes (Kyriakis and Avruch, 2001; Kyriakis and Avruch, 2012; Peti and Page, 2013). The activation of a MAPK cascade occurs in a module of consecutive phosphorylations, i.e., after a previous stimulus, each MAPK is phosphorylated by an upstream MAPKs. A MAPK module comprises a MAP3K that activates a MAP2K, which then, in turn, activates a MAPK (Pimienta and Pascual, 2007; Turjanski, Vaqué and Gutkind, 2007; Johnson, 2011; Kyriakis and Avruch, 2012; Peti and Page, 2013). MAPK phosphorylation events can be inactivated by MAPK protein phosphatases (MKPs) that dephosphorylate both phosphothreonine and phosphotyrosine residues on MAPKs (Liu et al., 2007; Pimienta and Pascual, 2007; Zhang and Dong, 2007).

**FIGURE 1:**
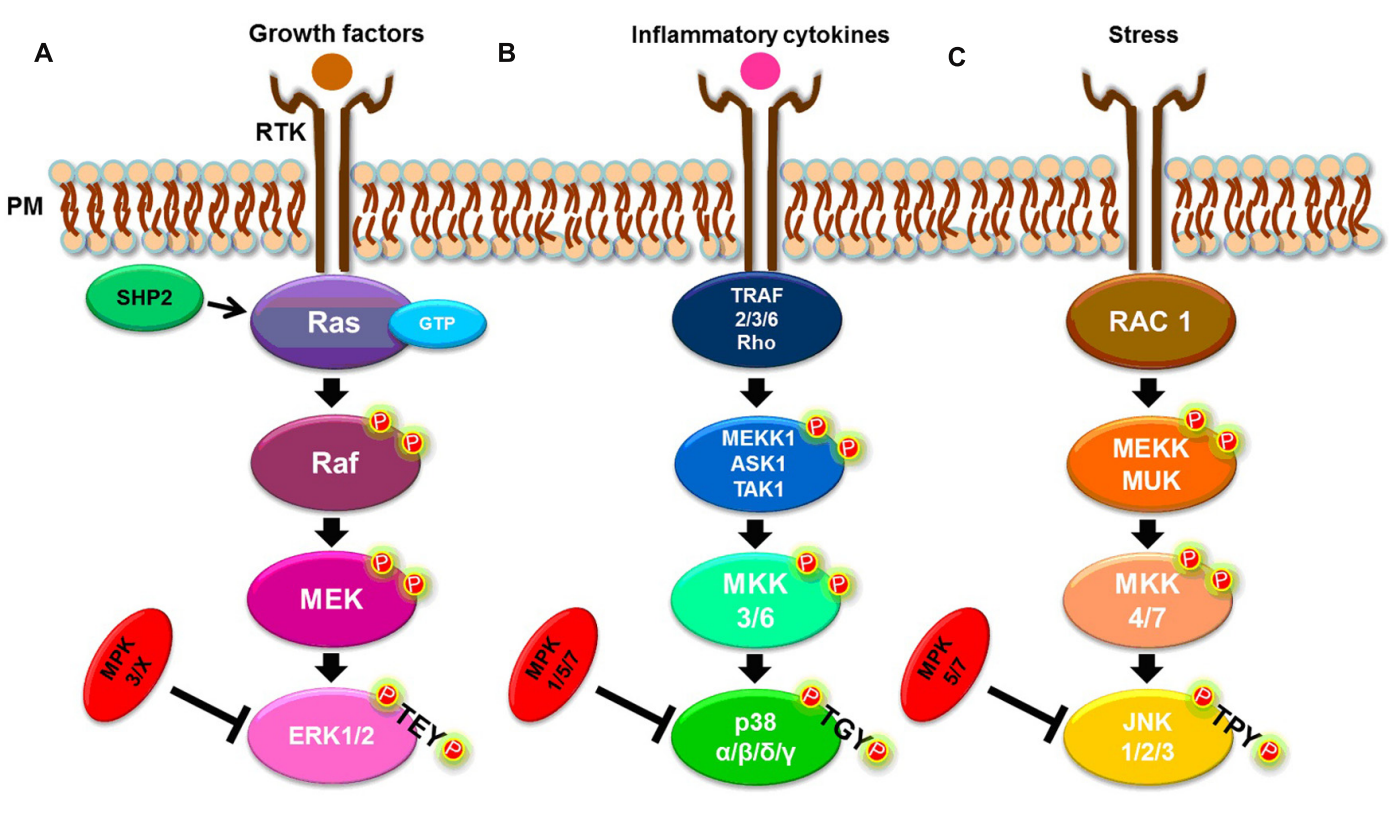
**Simplified MAPK signaling pathways.** **(A)** ERK1/2 pathway. **(B)** p38 α, β, δ, and γ pathways. **(C)** JNK 1, 2, and 3 pathways. See text for details.

There are three well-known MAPK pathways in mammalian cells (**Figure 1**): the ERK1/2, the c-JUN N-terminal kinase 1, 2 and 3 (JNK1/2/3), and the p38 MAPK α, β, δ, and γ pathways. ERK, JNK, and p38 isoforms are grouped according to their activation motif, structure and function (Owens and Keyse, 2007; Raman et al., 2007; Zhang and Dong, 2007). ERK1/2 is activated in response to growth factors, hormones and proinflammatory stimuli, while JNK1/2/3 and p38 MAPK α, β, δ, and γ are activated by cellular and environmental stresses, in addition to proinflammatory stimuli (Owens and Keyse, 2007; Kyriakis and Avruch, 2012; **Figure 1**).

## ERK and p38 MAPK Pathways

The classical activation of ERK1 and ERK2 isoforms is initiated by the binding of a ligand to a receptor tyrosine kinase (RTK) at the plasma membrane (PM), followed by activation of the small G-protein, Ras. In turn, Ras recruits and activates the serine/threonine protein kinase, Raf, a MAP3K, which activates the MAP2K, MEK, that, in turn, phosphorylates the MAPK, ERK1/2, at both threonine and tyrosine residues within the TEY motif (Kolch, 2000; Chambard et al., 2007; Shaul and Seger, 2007; Knight and Irving, 2014). The Ras/Raf/MEK/ERK1/2 pathway can be deactivated by dual-specificity MAPK phosphatases (MKPs). For example, MKP2/4 dephosphorylates ERK1/2, but can also deactivate other MAPKs while MKP3 and MKP-X are specific to ERK (Owens and Keyse, 2007). The tyrosine phosphatase, SHP2, also acts on this signaling pathway by activating the G-protein, Ras (Zhang et al., 2004; Matozaki et al., 2009; **Figure 1A**).

Both stress and cytokines activate p38 MAPK isoforms that play an important role in inflammatory responses (Johnson and Lapadat, 2002; Yang et al., 2014), despite each isoform being encoded by different genes and showing different tissue expression patterns (Cuadrado and Nebreda, 2010). As with ERK isoforms, p38 MAPKs are also sequentially activated. A canonical activation occurs when, in response to stress or cytokines, a MAP3K, such as MEKK1, ASK1, or TAK1, is activated by TRAF [TNF (tumor necrosis factor) receptor-associated factor] 2/3/6 or by Rho proteins. In turn, the MAP3K phosphorylates a MAP2K, either MKK3 or MKK6, that then phosphorylates the TGY motif of p38 isoforms (Cuenda and Rousseau, 2007; Cuadrado and Nebreda, 2010; **Figure 1B**). The p38 MAPKs α, β, δ and γ, are dephosphorylated by several dual-specificity protein phosphatases, such as MPK2/4, that can also deactivate ERK. The phosphatases, MPK5/7, can also dephosphorylate JNK and p38, while MPK1 exhibits a higher specificity for p38 (Owens and Keyse, 2007; Salojin and Oravecz, 2007).

## MAPKs and the Immune System

Many pathogens target host intracellular signaling pathways, including MAPK pathways, to inhibit immune responses (Roy and Mocarski, 2007; Arthur and Ley, 2013). The immune response is one of several key functions regulated by MAPKs, with the production of immunomodulatory cytokines, such as TNFα, interleukin (IL)-1, IL-10, and IL-12, induced by the activation of p38 MAPK, JNK, and ERK pathways (Dong et al., 2002; Arthur and Ley, 2013). The regulatory cytokines, IL-12 and IL-10, produced by specialized dendritic and macrophage cells, play an important role in the coordination of the immune response since IL-12 modulates the development of a Th1 response, which protects the host against intracellular parasites, and IL-10 promotes a Th2 response, which provides protection against extracellular infectious agents (Romagnani, 2006).

The production of IL-12 is regulated by p38 MAPK and, consequently, is involved in the induction of a Th1 response (Jackson et al., 2010; Arthur and Ley, 2013). Upon p38 MAPK activation and IL-12 production, Th cell differentiation is guided into a Th1-type cell that releases pro-inflammatory cytokines such as IL-2, IFN-γ, and TNF-α/β (Romagnani, 2006). Such cytokines mediate the immune response by acting to kill intracellular pathogens such as the protozoans, *Leishmania* spp. and *T. cruzi* (Beiting, 2014).

Conversely, the ERK1/2 pathway modulates the production of IL-10 (Chang et al., 2012) that induces Th cell differentiation into a Th2-type. In this manner, Th2 cells regulate the host’s humoral immune response by releasing anti-inflammatory cytokines, such as IL-4, IL-5, IL-9, and IL-13, that are involved in allergic reactions and the elimination of extracellular pathogens (Mosmann et al., 2009). Furthermore, IL-10 can also act as a negative regulator of inflammation to prevent tissue damage (Haddad et al., 2003; Romagnani, 2006; Guilliams et al., 2009; Bosschaerts et al., 2010).

## Host Immune Response Subversion by Protozoan Parasites

Protozoan parasites use many varied tactics to avoid and/or subvert the host’s immune response, with the adoption of an intracellular lifestyle one the first mechanisms (Sacks and Sher, 2002). Other successful strategies employed by such pathogens to circumvent the immune system include: the expression of specific parasite antigens on the surface of infected cells to prevent recognition by immune cells (Sacks and Sher, 2002), the subversion of T cell responses by interfering with cytokine production (Engwerda et al., 2014), and the avoidance of direct killing by the complement system (Ouaissi and Ouaissi, 2005).

To protect an organism against protozoan intracellular infection, its immune system needs to identify and eliminate the parasite, but, at the same time, also needs to be balanced in order to minimize or avoid self-inflicted tissue damage (Dent, 2002). Phagocytosis is the primary mechanism used by the immune system in its response to intracellular parasites. This mechanism, mainly promoted by macrophages, depends on parasite recognition and is enhanced by opsonization and the complement system (Stafford et al., 2002). *Leishmania* spp. and *T. cruzi* escape from the host’s complement system using different strategies. *T. cruzi* produces glycoproteins that inhibit C3b/C4b (gp160), factor B (gp58) and molecules that accelerate the decay of the C3 pathway. In contrast, *Leishmania* spp. adapt their membrane to prevent the insertion of C5b-C9. This species also produces a surface metalloproteinase, gp63, and a lipophosphoglycan (LPG) that cleave C3b, abrogating complement-mediated lysis (Sacks and Sher, 2002; Ouaissi and Ouaissi, 2005). *Leishmania* gp63 can also activate the complement system, leading to parasite opsonization and increased uptake by host macrophages in a highly advantageous mechanism that allows both forms of this parasite (promastigote and amastigote) to replicate inside host macrophages (Stafford et al., 2002; Gómez and Olivier, 2010).

Although phagocytosis is the first mechanism activated in immune protection, the induction of a Th1-type response is the most effective reaction against intracellular protozoans. The promotion of such an inflammatory response leads to the successful elimination of these parasites due to the release and intense activity of pro-inflammatory cytokines and mediators (Jankovic et al., 2001; Stafford et al., 2002; Guilliams et al., 2009; Bosschaerts et al., 2010; Beiting, 2014; Engwerda et al., 2014). As outlined previously, the generation of Th1 and Th2 responses is regulated by IL-12 and IL-10 cytokines, respectively, which are modulated by the host’s intracellular MAPK signaling pathways (Dong et al., 2002; Arthur and Ley, 2013). So, by acting on the MAPK signaling pathway, intracellular protozoan parasites can switch the production of regulatory cytokines from IL-12 to IL-10 to prevent the formation of an inflammatory response (Jankovic et al., 2001; Stafford et al., 2002; Zambrano-Villa et al., 2002; Guilliams et al., 2009). In this way, *T. cruzi* and *Leishmania* spp. impair the development of a Th1 response to favor a Th2-type response. The mechanisms used by these parasites to deregulate the immune response will be briefly reviewed below.

## *Leishmania* spp. Subvert the MAPK Pathway by Activating ERK1/2 to Increase IL-10 and Down-Regulate IL-12

*Leishmania* spp. parasites use several strategies to survive inside host cells after infection. The methods employed by these parasites to subvert the host’s immune defense systems include: (i) an intracellular stage in their life cycle, allowing them protection against humoral anti-leishmanicidal products; (ii) the suppression of the synthesis of reactive oxygen intermediates (ROI) or reactive nitrogen intermediates (RNI); (iii) the inhibition of antigen presentation by repressing the gene expression of major histocompatibility complex (MHC) class II, interfering with antigen loading, or by sequestering/highjacking the MHC II molecule or antigen; (iv) the subversion of host cellular signaling pathways such as STAT, PI3K/AKT, and MAPK; and (v) the modulation of host cytokines to avoid T cell differentiation and thus prevent the formation of a Th1-type immune response (Bogdan and Röllinghoff, 1998; Olivier et al., 2005; Liese et al., 2008; Martinez and Petersen, 2014).

To undermine the production of regulatory inflammatory cytokines and prevent the formation of a Th1-type immune response, *Leishmania* parasites target the MAPK signaling pathway, which is responsible for regulating the production of IL-12 (p38) and IL-10 (ERK 1/2) in macrophages and dendritic cells. By this means, *Leishmania* spp. promote switching from IL-12 to IL-10 production, consequently altering the formation of a Th1 response to Th2, and leading to parasite prevalence in the host (Ghalib et al., 1995; Olivier et al., 2005; Bhardwaj et al., 2010; Shadab and Ali, 2011).

**FIGURE 2:**
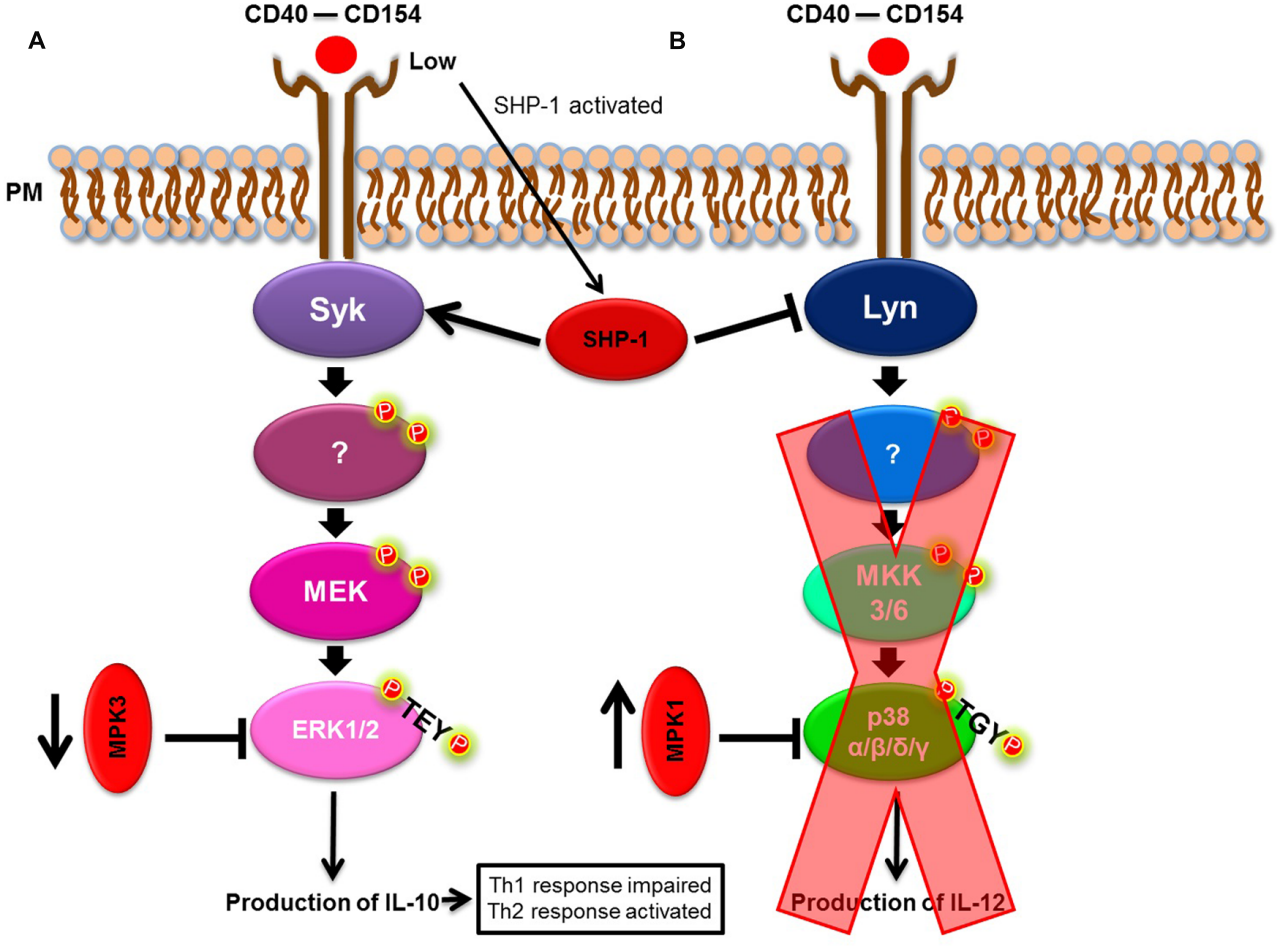
***Leishmania* spp. model of immune modulation targeting the p38/ERK1/2 MAPK pathway.** **(A)** CD40/ERK1/2 pathway. **(B)** CD40/p38 MAPK pathway. *Leishmania* spp. skew the CD40/MAPK signaling pathway, from p38 to ERK1/2, to favor IL-10 production.

Rub et al. (2009) have shown that *Leishmania* spp. often act upon the CD40/MAPK pathway. Expressed mainly on macrophages and dendritic cells, CD40 is an important co-stimulatory molecule involved in the differentiation of Th cells to a Th1-type, reflecting how CD40 induces the production of IL-12 (O’Sullivan and Thomas, 2003) by the activation of MAPK pathway members (Bhardwaj et al., 2010). In their study, Rub et al. (2009) showed that *L. major* triggered cholesterol depletion, and, in doing so, prevented CD40 reallocation to skew the CD40 signaling pathway from p38 and IL-12 to ERK1/2 and IL-10 production. As a consequence, this event led to an increased IL-10 production. Taken together, these events favored a parasite burden and confirmed that the CD40/MAPK pathway was important for *L. major* subversion of the host’s immune response. However, the specific mechanism surrounding augmented IL-10 production remained unknown until Srivastava et al. (2011) and Khan et al. (2014) noted the participation of phosphatases in this process. Srivastava et al. (2011) demonstrated that in *L. major* infection, the phosphatases, MKP-1 and MKP-3, were differentially expressed. *L. major* induced the upregulation of MKP-1 (p38 high affinity phosphatase) and downregulation of MKP-3 (ERK1/2 high affinity phosphatase) to skew CD40 signaling toward the ERK1/2 pathway, favoring infection. Khan et al. (2014) demonstrated that the phosphotyrosine phosphatase, SHP-1, functioned in a similar manner and acted on Syk and Lyn (ERK1/2 and p38 MAPK activators, respectively). In a CD40 dose-dependent manner, SHP-1 modulates CD40-induced phosphorylation of p38 MAPK and ERK1/2 to favor ERK1/2-dependent IL-10 expression and parasite survival (**Figure 2**). Xin et al. (2008) and Boggiatto et al. (2009, 2014) also demonstrated the importance of the CD40/MAPK signaling pathway in *L. amazonensis* infection. *L. amazonensis* upregulated ERK1/2 in dendritic cells, increased IL-10 production and prevented the expression of CD40 and IL-12p40 (one of the subunits of IL-12), leading to the limited activation of dendritic cells and a deficient Th1-type response.

Extracellular-signal-regulated kinase 1/2 activation by *Leishmania* spp., associated with parasite persistence and IL-12 down-regulation, was also seen for *L. amazonensis* (Xin et al., 2008; Martinez and Petersen, 2014) and for *L. donovani*. Infection by *L. donovani* also led to the suppression of p38 MAPK and increased IL-10 production (Chandra and Naik, 2008; Shadab and Ali, 2011). Interestingly, treatment of *L. donovani*-infected macrophages with an immunoprophylactic glycolipid, arabinosylated lipoarabinomannan (Ara-LAM) isolated from *Mycobacterium smegmatis*, activated p38 MAPK with a concomitant abrogation of ERK1/2 phosphorylation. Furthermore, the production of IL-12 (Bhattacharya et al., 2011) and IFNγ responsiveness were restored (Chowdhury et al., 2015). Exploitation of host phosphatases can also be seen in *L. donovani* infection: Nandan and Reiner (2005) showed that *L. donovani* activates the phosphotyrosine phosphatase, SHP-1 that correlates with parasite survival.

Gp63 and LPG can also modulate the host MAPK signaling pathway and the production of cytokines. Gp63 acts on both p38 and the phosphotyrosine phosphatase SHP-1. To subvert MAPK signaling, gp63 of *L. major* promastigotes leads to a p38 inhibition in fibroblasts by mediating the proteolysis of TAK-1-binding protein-1 (TAB1), a p38 regulator (Halle et al., 2009). Gp63 of both *L. major* and *L. mexicana* amastigotes and promastigotes activates SHP-1 in macrophages in a cleavage-dependent manner leading to p38 down-regulation (Gomez et al., 2009; Abu-Dayyeh et al., 2010). Gp63-mediated inactivation of p38 could consequently inhibit IL-12 production. Conversely, the LPG of *L. braziliensis* and *L. infantum* also activates ERK1/2, but abrogates not only IL-12 production, but also that of IL-10 (Ibraim et al., 2013). Further studies are needed to understand the mechanisms by which LPG modulates MAPK and IL-12 production in an IL-10-independent manner. *L. mexicana* LPG can also manipulate the MAPK pathway and inhibit IL-12 but this seems to be due to the impairment of NFκB translocation caused by LPG (Cameron et al., 2004; Argueta-Donohué et al., 2008; **Table 1A**).

**Table 1:** Trypanosomatids-released proteins and their action on MAPK pathway in macrophages and T cells.

(A) *Leishmania* spp. proteins				

**Protein**	**MAPK Target on macrophages**	**Mechanism**	**Target on T cells**	**Reference**
gp63	p38 MAPK	Cleavage-dependent activation of SHP-1 leading to p38 inactivation and, presumably, inhibition of IL-12	IL-12	Gomez et al., 2009; Abu-Dayyeh et al., 2010
LPG	ERK	Activation of ERK abrogating both IL-10 and IL-12 production	IL-10 and IL-12	Feng et al., 1999; Ibraim et al., 2013

**(B)** ***Trypanosoma cruzi* proteins**				

**Protein**	**MAPK Target on macrophages**	**Mechanism**	**Target on T cells**	**Reference**

Tc52	p38 or ERK	Mitogen-dependent modulation of genes that encode IL-10 and IL-12 leading to increased IL-10 secretion and inhibition of IL-12	IL-10 and IL-12	Ouaissi et al., 2002b; Borges et al., 2003; Ouaissi and Ouaissi, 2005
AgC10	p38	Inhibition of p38 and IL-12	IL-12	De Diego et al., 1997; Alcaide and Fresno, 2004
GPIs and GPI-anchored mucins	ERK	Activation of ERK1/2 upon treatment with GPIs and GPI-anchored mucins associated to IL-12 decrease	IL-12	Ropert et al., 2001
TS	ERK	TS activates ERK and stimulates IL-10 secretion	IL-10	Chuenkova and Pereira, 2001; Ruiz Díaz et al., 2015


## *Trypanosoma cruzi* Triggers Molecules to Regulate the MAPK Pathway and Cytokine Production

The mechanisms by which *T. cruzi* evades the host’s immune response by acting on the MAPK pathway have been poorly studied and are therefore incompletely understood. However, it is well established that upon cell invasion, *T. cruzi* begins to subvert signaling pathways and to use host molecules to favor its entry and survival inside host cells. For instance, *T. cruzi* extracellular amastigotes (EAs) recruit both host protein kinase D1 (PKD1) and cortactin to induce PKD1 autophosphorylation and cortactin activation by ERK, leading to the recruitment of host actin that allows parasite entry into HeLa cells (Bonfim-Melo et al., 2015). *T. cruzi*, as well as *Leishmania* spp., is able to induce: (i) ERK1/2, but not p38 MAPK, activation in macrophages and dendritic cells (Mukherjee et al., 2004); and (ii) increased IL-10 and decreased IL-12 production (Poncini et al., 2008). These effects impair the formation of an efficient Th1 inflammatory response (Alba Soto et al., 2003, 2010) to allow parasite evasion of the host immune response.

Some *T. cruzi* molecules are released and activate Toll-like receptors (TLRs), such as TLR2, TLR4, or TLR9, in dendritic cells and macrophages (Tarleton, 2007). This leads to the activation of p38 MAPK and the production of IL-12, favoring an inflammatory response (Ropert et al., 2001; Terrazas et al., 2011). This model is supported by the observations of Terrazas et al. (2011) who showed that dendritic cells exposed to *T. cruzi* antigens (TcAgs) and TLR ligands induced p38 phosphorylation that was dependent on TcAg-macrophage migration inhibitory factor (MIF) synergism. This led to the enhancement of IL-12 production, thus promoting a Th1-type response (Terrazas et al., 2011).

However, despite the activation of a pro-inflammatory immune response by some parasite molecules, it is well known that several other molecules of such pathogens act against the host’s infected cells and signaling pathways, subverting the host’s immune response against the parasite (Ouaissi et al., 1995; Hovsepian et al., 2011; Castillo et al., 2013; Ruiz Díaz et al., 2015).

One of the proteins released by *T. cruzi* that disrupts the host’s immune response is Tc52. A protein of 52 kDa, Tc52 is composed of two homologous domains sharing significant homology with glutathione *S*-transferases (Schöneck et al., 1994), and exhibits both immunomodulatory and virulence roles (Ouaissi et al., 1995, 2002a). When localized in the cytoplasm, a 28 kDa peptide fragment derived from the C-terminal portion of Tc52 (Borges et al., 2003) induces the Tc52-mediated suppression of T cell proliferation, and exerts mitogen-dependent cytokine and chemokine-like activities. Thus, this peptide modulates genes that encode IL-10 and IL-12, leading to increased IL-10 secretion and thereby inhibiting IL-12 (Ouaissi et al., 2002b; Ouaissi and Ouaissi, 2005). Moreover, the events outlined above are probably mediated by MAPKs.

Other proteins related to immune modulation in *T. cruzi* infection are the glycosylphosphatidylinositol (GPI)-anchored mucins and *trans*-sialidases (TS). AgC10, a GPI-anchored mucin of 40–50 kDa, inhibits TNF and IL-12 secretion in a p38 MAPK inhibition-dependent manner, impairing the formation of a Th1 response (De Diego et al., 1997; Alcaide and Fresno, 2004). Ropert et al. (2001) reported that ERK1/2 activation was associated with a decrease in IL-12 in macrophages treated with *T. cruzi* GPI and GPI-mucins, corroborating the participation of these proteins in modulating the host’s immune response. However, they also showed that GPIs and GPI-anchored mucins could activate p38 MAPK later than ERK1/2, thus increasing IL-12 synthesis and generating an opposing effect in the regulation of the immune response by promoting a Th1 response (Ropert et al., 2001). *T. cruzi* TS have been linked to ERK1/2 activation (Chuenkova and Pereira, 2001). Recently, Ruiz Díaz et al. (2015) confirmed the role of TS in IL-10-stimulated secretion, leading to an imbalance of the Th1 cell response toward an Th2 phenotype (**Table 1B**). However, despite current knowledge concerning the strategies used by *T. cruzi* to subvert the host’s immune response, the precise mechanisms by which this occurs remain unknown.

## Conclusion

The MAPK signaling pathway, responsible for regulating the production of Th1-and Th2-type responses, is targeted by trypanosomatids to modulate the host’s immune response in order to favor parasite replication and survival. The mechanisms whereby *Leishmania* spp. skew the MAPK signaling pathway to subvert cytokine production and switch a Th1 to a Th2 response are well known compared to those for *T. cruzi*. *Leishmania* parasites often target the CD40/MAPK pathway, activating ERK1/2 to increase and decrease IL-10 and IL-12 production, respectively. In comparison, the strategy used by *T. cruzi* is to trigger molecules to subvert MAPK ERK1/2 and p38 pathways and thus modulate cytokine production. Further studies are required to increase our understanding of the intriguing mechanisms by which *T. cruzi* manipulates the host’s immune response.

## Author Contributions

MS-S, FD and GG contributed equally to the writing of the review. DB conceived and wrote the review.

## Conflict of Interest Statement

The authors declare that the research was conducted in the absence of any commercial or financial relationships that could be construed as a potential conflict of interest.
